# High‐throughput screening of clinically approved drugs that prime polyethylenimine transfection reveals modulation of mitochondria dysfunction response improves gene transfer efficiencies

**DOI:** 10.1002/btm2.10017

**Published:** 2016-07-21

**Authors:** Albert Nguyen, Jared Beyersdorf, Jean‐Jack Riethoven, Angela K. Pannier

**Affiliations:** ^1^ Dept. of Biological Systems Engineering University of Nebraska‐Lincoln Lincoln NE 68583; ^2^ Center for Nanohybrid Functional Materials University of Nebraska‐Lincoln, Lincoln NE 68588; ^3^ Dept. of Electrical and Computer Engineering University of Nebraska‐Lincoln Lincoln NE 68588; ^4^ Bioinformatics Core Research Facility University of Nebraska‐Lincoln Lincoln NE 68588; ^5^ School of Biological Sciences University of Nebraska‐Lincoln, Lincoln NE 68588

**Keywords:** high‐throughput screen, mitochondrial dysfunction, NIH Clinical Collection, nonviral gene delivery, polyethylenimine toxicity, priming transfection

## Abstract

Nonviral gene delivery methods are advantageous over viral vectors in terms of safety, cost, and flexibility in design and application, but suffer from lower gene transfer efficiency. In addition to modifications to nucleic acid design and nonviral carriers, new tools are sought to enhance transfection. Priming is the pharmacological modulation of transfection efficiency and transgene expression, and has demonstrated transfection increase in several compounds, for example, chloroquine and glucocorticoids. To develop a library of transfection priming compounds, a high‐throughput screen was performed of the NIH Clinical Collection (NCC) to identify clinical compounds that prime polyethylenimine (PEI) transfection. HEK293T cells were treated with priming compounds, then transfected with enhanced green fluorescent protein (EGFP)‐encoding plasmid by PEI. After 48‐hr culture, primed and transfected cells were assayed for transfection, cell proliferation, and cell viability by fluorescence measurement of EGFP reporter, Hoechst 33342 nuclei stain, and resazurin metabolic assay. From the microscope image analysis and microplate measurements, transfection fold‐changes were determined, and compounds resulting in statistically significant transfection fold‐change were identified. NCC compounds were clustered using PubChem fingerprint similarity by Tanimoto coefficients in ChemmineTools. Fold‐changes for each compound were linked to drug clusters, from which drug classes that prime transfection were identified. Among the identified drugs classes that primed transfection increases were antioxidants, GABAA receptor modulators, and glucocorticoids. Resveratrol and piceid, stilbenoid antioxidants found in grapes, and zolpidem, a GABAA modulator, increased transfection nearly three‐fold. Literature indicate interaction of the identified transfection priming drug clusters with mitochondria, which may modulate mitochondrial dysfunction known to be associated with PEI transfection.

## 1 Introduction

Gene delivery, the transfer of exogenous nucleic acids into cells to modify gene expression, can be accomplished with either viral or nonviral methods. Viral transduction is highly efficient in vitro and in vivo, however, transduction is limited by design constraints and safety concerns, a few of which include: size of transgene,^1^ host immune response,^2^ and insertional mutagenesis.^3^ Nonviral gene delivery methods often make use of chemical strategies that rely on cationic carriers, which can associate with nucleic acids to form complexes that achieve gene delivery.^1^ These materials offer advantages over viral methods of gene delivery, in safety, cost, fabrication, and flexibility in design and application. However, compared to viral methods, chemical methods of nonviral gene delivery suffer from lower transfection efficiency, especially in vivo.^4^ Many barriers to successful transfection exist, with each contributing to the overall low efficiency of nonviral gene delivery. Systemic barriers include serum stability, clearance, and targeting,^5^ while cellular barriers to efficient transfection include cellular internalization, endosomal escape, nuclear transport, nuclear import, and transcriptional and translational regulation.^5^

Carrier properties and optimization are the most important determinants in the efficiency of chemical nonviral gene delivery strategies in terms of facilitating delivery to and into cells. In 1995, Polyethylenimine (PEI), a cationic polymer, demonstrated successful transfection in vitro and in vivo in multiple cell types.^6^ General optimization of PEI transfection is accomplished through tuning of N:P ratio, the ratio of amine groups in the PEI polymer to phosphate groups in the nucleic acid. N:P ratio determines the overall size and charge of these PEI‐nucleic acid complexes and defines their ability to circulate in serum, internalize into cells, and escape endosomes, and thus are primary determinants of PEI transfection efficiency.^6^, ^7^, ^8^ Optimized PEI transfection achieves efficiency of 50–80% in vitro depending on molecular weight of PEI, plasmid, and cell type,^8^ compared to the 100% efficiency achieved by some optimized viral transduction methods in vitro.^9^ Most attempts to improve chemical methods of nonviral gene delivery involve modification of the carriers, such as modification of PEI with PEG and targeting ligands^8^ to increase serum stability and circulation or enhance specific uptake, respectively. Although nonviral gene delivery methods continue to be improved, they have not exceeded viral methods in gene transfer efficiency and sustained expression in clinical trials.^10^

We propose pharmacological priming as an adjuvant strategy, which can, in conjunction with innovations in carrier and nucleic acid design, achieve clinically relevant nonviral gene transfer efficiency and expression. Priming refers to the treatment of cells with chemical compounds before, during, or after delivering carrier‐nucleic acid complexes to cells in order to improve some aspect of the gene transfer process. Priming enhancement of transfection efficiency and transgene expression can be achieved through direct modulation of the barriers to gene delivery, or indirectly through modulation of the cellular response to transfection in terms of toxicity and altered gene expression.

Priming compounds for gene delivery have been used in the literature, mostly in the context of probing biological mechanisms of transfection. For example, chloroquine is an antimalarial compound that has been demonstrated to enhance nonviral gene delivery in transfections across several nanoparticle carrier formulations,^11^ through buffering of endosomes, aiding complexes in escaping endosomes and avoiding lysosomal degradation.^11^, ^12^ Glucocorticoid priming has been demonstrated to enhance viral and nonviral gene delivery across multiple cell types.^13^, ^14^, ^15^ Recent work showed that priming human mesenchymal stem cells (hMSCs), a primary cell type that is typically difficult to transfect, with dexamethasone (a synthetic glucocorticoid), increased transfection efficiency and transgene expression up to 10‐fold across multiple donors.^15^ Furthermore, pharmacological priming, with activators and inhibitors of genes previously identified as differentially expressed in successfully versus unsuccessfully transfected HEK293T cells, was shown to enhance or decrease transgene expression,^16^, ^17^, ^18^, ^19^ demonstrating that genomic targets can be modulated by priming to enhance nonviral gene delivery. Several of these genes were related to cell stress, indicating that in addition to overcoming the primary barriers to transfection, nonviral gene delivery strategies may need to modulate the overall cellular response to transfection to achieve higher transfection efficiency and more sustained transgene expression.

While broad applicability of transfection priming to different carriers, nucleic acids, and cell types has not been established, a next step in the development of this strategy is to search for other compounds that have priming effects. Priming strategies can theoretically be used to modulate any of the known barriers to transfection, from transport mechanisms into the cell and to the nucleus, to transcriptional and translational regulation and toxicity. To broadly search for potential priming mechanisms, a high‐throughput, drug repurposing approach was taken toward generating a library of compounds from the NIH Clinical Collection (NCC)^20^ that possess clinically relevant bioavailability and biocompatibility, and whose priming effects could be studied and used to develop effective nonviral gene delivery strategies.

## 2 Materials and Methods

### 2.1 HEK293T culture and transfection reagents

HEK293T cells (ATCC, Manassas, VA) were cultured in T‐25 flasks (Thermo Fisher Scientific, Waltham, MA) in Dulbecco's Modified Eagle Medium (DMEM) (Life Technologies, Carlsbad, CA) completed with 10% fetal bovine serum (Life Technologies), 1% Penicillin/Streptomycin (Life Technologies), and 1% sodium pyruvate (Life Technologies). Cells were incubated at 37°C, 5% CO_2_, and passaged using 1 mM EDTA in PBS (Sigma‐Aldrich, St. Louis, MO) at 75–85% confluence approximately every 48 hrs.

The pEGFPLuc plasmid delivered in this transfection screen encodes for a fusion protein of enhanced green fluorescent protein (EGFP) and luciferase, driven by a CMV promoter (Clontech, Mountain View, CA). pEGFPLuc was propagated in *E. coli*, selected by kanamycin treatment, isolated using Qiagen Giga Prep kits (Qiagen, Valencia, CA), prepared in endotoxin‐free TE buffer at 1 mg/ml, and subsequently stored in frozen aliquots at −20°C. PEI (25 kDa branched PEI, Sigma‐Aldrich) was used for this transfection screen. PEI was prepared at 1 mg/ml, in 0.1 M sodium bicarbonate (pH 8.2), then stored in aliquots at −80°C.

### 2.2 Priming compounds and fluorescence assay reagents

The NIH NCC, which contains 725 compounds screened for transfection priming, were received in ten, 96‐well plates, with 10 μl of each compound at 10 mM in dimethyl sulfoxide (DMSO). To prepare aliquots, 2 μl of each compound were prepared in four 96‐well aliquot plates per original NCC plate, and kept frozen at −20°C. Hoechst 33342 fluorescent dye was used to stain cell nuclei and enable cell‐count by processing of fluorescence microscope images, while resazurin was used as a fluorescent metabolic indicator in this screen. For cell staining, Hoechst 33342 stock solution was prepared from lyophilized powder (Sigma‐Aldrich), dissolved in ddH_2_0 at a concentration of 10 mg/ml, and stored at 4°C. Stock resazurin solution was prepared from lyophilized resazurin sodium salt powder (Sigma‐Aldrich), dissolved in PBS at a concentration of 10 mg/ml, and stored at 4°C.

### 2.3 Transfection priming high‐throughput screen

HEK293T cells were detached from culture flasks by EDTA, counted by hemocytometer, and seeded at 25,000 cells/cm^2^ (8,000 cells per well in 80 μl) into 96‐well plates. Four plates were seeded for each of the 10 plates of the NCC collection screened, for duplicate testing of the compounds at 5 and 50 μM. Seeded plates were cultured at 37°C, 5% CO_2_, and approximately 17 hr after seeding two plates with cells, 40 μl of each NCC compound were delivered into corresponding wells of both seeded plates, at either 5 or 50 μM final well volume concentration. Two columns (16 wells) of each plate received equivalent DMSO % instead of NCC compound as priming vehicle controls. The primed plates were then incubated for one hour at 37°C, 5% CO_2_ before transfection. For transfection, PEI was diluted in 1X Tris‐buffered saline (TBS) and added to pEGFPLuc diluted in 1X TBS. Complexes were formed at an optimized (data not shown) N:P of 15 to deliver 0.17 μg of pEGFPLuc in 21 μl of TBS to each well 1 hr after priming, followed by incubation at 37°C, 5% CO_2_ for 48 hr before staining and imaging.

The primed and transfected HEK293T plates were stained with Hoechst and resazurin fluorescent dyes to enable subsequent nuclei counts and viability assessments, respectively, multiplexed with the EGFP fluorescent reporter for transgene expression. Staining solution consisted of 17.5 μg/ml Hoechst 33342 and 10 μg/ml resazurin in Fluorobrite DMEM (Thermo Fisher Scientific). After rinsing cells with PBS, 100 μl of the staining solution was added to each well, followed by incubation for 30 min at 37°C, 5% CO_2_.

After incubation with the staining solution, plates were imaged using a DMI3000B manual inverted microscope with DFC340FX digital camera, EL‐6000 mercury halide lamp for fluorescence excitation, and LAS software V4.0 for digital image viewing and capture (Leica, Buffalo Grove, IL). Filters for excitation and detection at the EGFP and Hoechst wavelengths (488 nm/509 nm and 355 nm/465 nm, respectively) were used to take monochrome images of their fluorescence, in addition to phase contrast images. Images were taken with a 5x objective at the center of each well in 8‐bit TIFF format and 1600x1200 pixels, with each pixel having a greyscale value from 0 to 255. Consistent fluorescence excitation lamp intensity and camera exposure settings were used to allow for comparison of image intensities between wells in the same plate.

Macros in ImageJ,^21^ a Java‐based image processing program, were used to automate the mean fluorescence intensity measurements and maxima counts for the processing of the thousands of images acquired over the course of the screen. Average grey values of the Hoechst and EGFP images were determined using the “Measure” plugin in ImageJ. Total cells and transfected cells were counted for each well using the “Find Maxima” plugin to count Hoechst stained nuclei and EGFP expressing cells, respectively. The noise tolerance of the “Find Maxima” plugin was adjusted to optimize detection and minimize misidentification of nuclei and EGFP cells.

In addition to microscope imaging, EGFP, Hoecshst, and resazurin fluorescence were measured by Synergy H1 plate reader (BioTek, Winooski, VT), with excitation/emission settings of 475 nm/509 nm, 355 nm/464 nm, and 545 nm/590 nm, and gain settings of 100, 50, and 50, respectively. Nine measurements were taken per well in a three by three array equally spaced within the well, from which mean intensities were calculated.

### 2.4 Data processing

The goal of the data processing was to determine compounds that produced fold‐changes in transfection, compared to vehicle controls. To reduce potential fold‐change bias resulting from initial seeding density variation, vehicle control wells that had total cell‐counts less than the mean cell‐count of vehicles controls as a whole for the plate were discarded from consideration for calculations of fold‐changes. There remained up to 16 vehicle control wells per plate in the screen after filtering (*n* ≤ 16). Image and plate reader data for each well were then normalized by cell‐count and their fold‐changes determined relative to these filtered vehicle control wells. EGFP measurements were normalized into two separate measures: normalization by total cell‐count and normalization by transfected cell‐count. Hoechst and resazurin measurements were normalized by total cell‐count.

Cytotoxicity filters were implemented to remove from consideration compounds that were too toxic at the tested concentrations to warrant further consideration in the screen. In the first toxicity filter, compounds with normalized Hoechst‐count fold‐change of less than 0.2 were discarded. In the second toxicity filter, compounds with Hoechst‐count fold‐change two or three standard deviations below the plate‐wide average of Hoechst‐count fold‐changes greater than 0.2 were also discarded. A more stringent toxicity filter was used for the 5 μM concentration (two standard deviations) than the 50 μM (three standard deviations). The rationale behind this difference is that the 5 μM conditions contain a lower concentration of priming compound and of the DMSO vehicle, resulting in less cytotoxicity than the 50 μM conditions; therefore, a more stringent filter was applied to the lower concentration.

Each compound was tested in a single well per plate, in duplicate plates, at 5 and 50 μM concentrations. The duplicate normalized fold‐changes of these single wells from duplicate plates were grouped (*n* = 2) for unpaired one‐tailed *t* tests against grouped filtered vehicle controls from the same duplicate plates (*n* ≤ 32 filtered vehicle control wells from the duplicate plates associated with the tested compound of *n* ≤ 16 control wells each), to obtain *p* values for EGFP, Hoechst, and resazurin measurements of each compound at each concentration, with an α‐value for significance of 0.05. For each well, the assumption of equal or unequal variance in the *t* test was estimated by *F*‐test (one‐tailed, α = 0.05) for equality of variances between tested compound and control wells. One‐tailed *t* tests were used to score the screen fold‐changes, in spite of expected higher false positive rates than two‐tailed tests, to err on the side of caution in not rejecting potential priming compounds, and rely on the clustering results to support the single compound hit selection as well as future investigation of the screen hits to reject false positives.

Given the large number of drugs to be tested in the NCC, we chose to use a sample size of two for each concentration of each drug. Two is the minimum number of samples for performing a *t* test^22^; more importantly, the goal of this screen was to be the initial step in the search for clinical compounds that could be repurposed toward transfection priming, using a high throughput methodology to deliberately test in small sample sizes, sacrificing power for breadth of small molecules tested from the NCC.

### 2.5 Hit selection and compound clustering

All NCC compounds that exhibited significant fold‐increases or fold‐decreases in transfection, at either 5 or 50 μM, and were not filtered for cytotoxicity, were identified as hits (α‐value for significance of 0.05). In addition, the hit selection of this screen also included examination of overall transfection fold‐changes of grouped drug clusters to identify drug classes for which the majority of compounds were hits for transfection priming effects. NCC compounds were clustered using the Chemmine Tools web platform for comparison of PubChem fingerprints by Tanimoto coefficients.^23^ Hierarchical clustering was used to create a dendrogram based on single linkage fingerprint similarity, while binning clustering grouped compounds based on single linkage fingerprint similarity determined by Tanimoto coefficient thresholds of 0.4 to 0.9 (higher coefficient indicates greater fingerprint similarity).

EGFP, Hoechst, and resazurin measurement fold‐changes for each compound at each concentration were linked to the NCC clusters by a custom Perl script, which parsed the data files and grouped the data into average fold‐changes for each binned cluster. To analyze the hits, the compounds which primed the 10 highest fold‐increases or fold‐decreases in transfection at either 5 or 50 μM were determined (Tables 1 and 2), which were used to determine clusters of interest (Figure 2). The grouped fold‐changes in transfection of the clusters of interest were examined to determine clusters for which the majority of member compounds were hits for priming transfection (Table 3).

**Table 1:** Highest fold‐changes in transfection priming hits at 5mM of NCC compounds

	Compound	Drug type	Transfection^a^	Cell‐count^b^	Hoechst‐intensity^c^	Resazurin‐intensity^d^
Transfection fold‐increase	Zolpidem tartrate	GABA receptor modulator	3.05	0.52		
	Resveratrol	Stilbenoid	3.04	0.55		
	Tropisetron hydrochloride	Serotonin receptor antagonist	2.67	0.97	0.91	1.07
	Tranilast	Anti‐allergy	2.17	0.74	1.11	1.29
	Lansoprazole	Proton‐pump inhibitor	2.13	0.72	1.13	1.42
	Nobiletin	Flavonoid	2.08	0.61	1.23	1.21
	Nitrazepam	GABA receptor modulator	2.03	0.53		
	Enalaprilat	Angiotensin‐converting‐enzyme inhibitor pro‐drug	2.00	0.55	1.35	1.51
	Droperidol	Dopamine receptor antagonist	1.95	0.52		
	Mestanolone	Androgen hormone	1.93	0.61	1.26	
Transfection fold‐decrease	Epigallocatechin gallate	Flavonoid	0.08	1.37	0.88	
	Ampiroxicam	Nonsteroidal anti‐inflammatory drug	0.31	0.93	0.91	1.14
	Nimodipine	Calcium channel blocker	0.35	0.90		
	(−)‐Cotinine	Nicotine metabolite	0.41	0.87		1.16
	Ramipril	Angiotensin‐converting‐enzyme inhibitor	0.42	0.98	0.92	
	Desloratadine	Anti‐histamine	0.50	0.86		1.19
	Crotamiton	Anti‐itch	0.50	0.98	0.93	
	Guanidine	Amino acid metabolite	0.52	0.93		1.12
	Letrozole	Estrogen synthesis inhibitor	0.52	1.00	0.87	
	Fluphenazine hydrochloride	Dopamine receptor antagonist	0.53	0.85	1.08	1.17

*Note*. Empty values indicate nonsignificant fold‐change.

a Transfection fold‐changes were calculated from duplicate averages of EGFP fluorescence, measured by EGFP cell‐count (image processing) or EGFP intensity (plate reader and image processing), relative to the same measurement averaged from the vehicle controls in each compound's respective plates. These measurements were normalized in two separate ways, total cell‐count (determined by Hoechst‐count), and transfected cell‐count (determined by EGFP‐count), for measurement of transfection per cell as well as transfection per transfected cell.

b Cell‐count fold‐changes were calculated from duplicate averages of image processing measurements of Hoechst‐count relative to the average Hoechst‐count measurements of vehicle controls in each compound's respective plates. Cell‐count fold‐changes are shown for each hit presented as a general toxicity reference, not to imply significant increase or decrease.

c Hoechst‐intensity fold changes were calculated from duplicate averages of plate reader or image processing measurements of Hoechst‐intensity normalized by total cell‐count (determined by Hoechst‐count), relative to the average Hoechst‐intensity measurements of vehicle controls in each compound's respective plates.

d Resazurin‐intensity fold changes were calculated from duplicate averages of plate reader measurements of resazurin intensity normalized by cell‐count (determined by Hoechst‐count), relative to the average resazurin‐intensity measurements of vehicle controls in each compound's respective plates.

**Table 2:** Highest fold‐changes in transfection priming hits at 50 mM of NCC compounds

	Compound	Drug type	Transfection^a^	Cell‐count^b^	Hoechst‐intensity^c^	Resazurin‐intensity^d^
Transfection fold‐increase	Tranilast	Anti‐allergy	3.63	0.55		1.24
	Piceid	Stilbenoid	2.63	0.69	1.17	
	5‐Fluorocytosine	Pyrimidine analogue	2.54	0.52	1.50	1.58
	Cinanserin	Serotonin receptor antagonist	2.53	0.63	1.41	
	Zardaverine	Phosphodiesterase inhibitor	2.26	0.55	1.36	
	Nateglinide	ATP potassium channel closer	2.08	0.51	1.23	1.21
	Eryped	Macrolide antibiotic	1.99	0.72	1.26	1.26
	Mestinon	Cholinesterase inhibitor	1.83	0.77	1.21	1.23
	Acyclovir	DNA polymerase inhibition (anti‐viral)	1.78	0.60		
	Stiripentol	GABA receptor modulator	1.77	0.72	1.23	
Transfection fold‐decrease	Epigallocatechin gallate	Flavonoid	0	0.80		
	Cefixime trihydrate	β‐lactam antibiotic	0.017	1.45	0.84	
	Cefdinir	β‐lactam antibiotic	0.032	1.35	0.88	
	Cefuroxime	β‐lactam antibiotic	0.039	1.39		
	Rolitetracycline	Tetracycline antibiotic	0.063	1.42	0.86	
	Cefatrizine propylene glycol	β‐lactam antibiotic	0.095	1.75	0.78	
	Tetracycline	Tetracycline antibiotic	0.139	1.08	0.85	
	Taxifolin‐(+)	Flavonoid	0.162	1.22	0.87	
	(+/−)‐Epinephrine hydrochloride	Adrenergic receptor agonist	0.35	1.36	0.83	
	IsoquercitrinHyperoside	FlavonoidFlavonoid	0.358	1.68	0.86	

*Note*. Empty values indicate nonsignificant fold‐change.

a Transfection fold‐changes were calculated from duplicate averages of EGFP fluorescence, measured by EGFP cell‐count (image processing) or EGFP intensity (plate reader and image processing), relative to the same measurement averaged from the vehicle controls in each compound's respective plates. These measurements were normalized in two separate ways, total cell‐count (determined by Hoechst‐count), and transfected cell‐count (determined by EGFP‐count), for measurement of transfection per cell as well as transfection per transfected cell.

b Cell‐count fold‐changes were calculated from duplicate averages of image processing measurements of Hoechst‐count relative to the average Hoechst‐count measurements of vehicle controls in each compound's respective plates. Cell‐count fold‐changes are shown for each hit presented as a general toxicity reference, not to imply significant increase or decrease.

c Hoechst‐intensity fold changes were calculated from duplicate averages of plate reader or image processing measurements of Hoechst‐intensity normalized by total cell‐count (determined by Hoechst‐count), relative to the average Hoechst‐intensity measurements of vehicle controls in each compound's respective plates.

d Resazurin‐intensity fold changes were calculated from duplicate averages of plate reader measurements of resazurin intensity normalized by cell‐count (determined by Hoechst‐count), relative to the average resazurin‐intensity measurements of vehicle controls in each compound's respective plates.

**Table 3:** Average fold‐changes^a^ of transfection priming hits from clusters of interest (Figure 2)

		Transfection fold‐increase hits			Transfection fold‐decrease hits		
Cluster (#)	Concentration	# of hits	Transfection^b^	Cell‐count^c^	# of hits	Transfection^b^	Cell‐count^c^
Hormones (54)	5 µM	34*	1.43 ± 0.22	0.73 ± 0.15	3	0.81 ± 0.07	0.93 ± 0.19
	50 µM	15	1.35 ± 0.16	0.66 ± 0.86	9	0.74 ± 0.12	0.84 ± 0.24
Glucocorticoids (30)	5 µM	17*	1.37 ± 0.14	0.77 ± 0.14	3	0.81 ± 0.07	0.93 ± 0.19
	50 µM	12	1.37 ± 0.15	0.67 ± 0.10	3	0.81 ± 0.04	1.01 ± 0.08
Tetracyclines (6)	5 µM	5*	1.27 ± 0.15	0.82 ± 0.06	1	0.91	0.72
	50 µM	0			3*	0.26 ± 0.27	1.07 ± 0.36
Fluoroquinolones (8)	5 µM	5*	1.54 ± 0.24	0.64 ± 0.17	0		
	50 µM	4	1.56 ± 0.25	0.63 ± 0.11	2	0.65 ± 0.21	0.96 ± 0.09
β‐lactams (18)	5 µM	7	1.25 ± 0.17	0.93 ± 0.13	2	0.69 ± 0.12	1.26 ± 0.14
	50 µM	5	1.25 ± 0.11	0.96 ± 0.20	9*	0.42 ± 0.37	1.31 ± 0.21
Cephalosporins (10)	5 µM	4	1.33 ± 0.19	0.85 ± 0.09	1	0.61	1.36
	50 µM	1	1.19	1.08	7*	0.31 ± 0.35	1.35 ± 0.22
Macrolides (4)	5 µM	3*	1.24 ± 0.05	0.82 ± 0.19	0		
	50 µM	2*	1.72 ± 0.39	0.63 ± 0.12	2*	0.74 ± 0.18	0.85 ± 0.42
Flavonoids and Stilbenoids (13)	5 µM	3	2.1 ± 0.86	0.72 ± 0.37	4	0.52 ± 0.33	1.04 ± 0.51
	50 µM	3	2.04 ± 0.61	0.69 ± 0.09	4	0.32 ± 0.32	1.26 ± 0.36
GABA modulators (9)	5 µM	7*	1.74 ± 0.63	0.66 ± 0.14	0		
	50 µM	4	1.41 ± 0.25	0.72 ± 0.04	0		

*Note*. Nobileton, a flavonoid (Table 1), was not automatically grouped into the flavonoid and stilbenoid cluster (Figure 2) by the clustering algorithm; it was manually added to the flavonoid and stilbenoid cluster for the calculation of cluster average fold‐changes. Similarly, stiripentol (Table 2) and zolpidem (Table 1) are GABAA receptor modulators which were not automatically clustered with the benzodiazepines (Figure 2); their fold‐changes were included into the GABAA modulator cluster for calculation of cluster average fold‐changes (Table 3).

a Average fold‐changes are shown as mean ± standard deviation.

b Transfection fold‐changes of hits from the same cluster were averaged. The transfection fold‐changes of hits were calculated from duplicate averages of EGFP fluorescence, measured by EGFP cell‐count (image processing) or EGFP intensity (plate reader and image processing), relative to the same measurement averaged from the vehicle controls in each compound's respective plates. These measurements were normalized in two separate ways, total cell‐count (determined by Hoechst‐count) and transfected cell‐count (determined by EGFP‐count), for measurement of transfection per cell as well as transfection per transfected cell.

c Cell‐count fold‐changes of hits from the same cluster were averaged. The cell‐count fold‐changes were calculated from duplicate averages of image processing measurements of Hoechst‐count relative to the average Hoechst‐count measurements of vehicle controls in each compound's respective plates. Average cell‐count fold‐changes are shown for clustered hits as a general toxicity reference, not to imply significant increase or decrease.

*The majority of compounds in the cluster were hits for this fold‐change.

^#^Denotes the number of NCC compounds in the cluster.

### 2.6 Preliminary verification experiment

Preliminary verification experiments were performed for three of the priming compounds identified (resveratrol, epigallocatechin gallate [EGCG], and corticosterone), testing these hit compounds at 5 μM in triplicate in 48‐well plates, with number of seeded cells and volumes of reagents added scaled by the well surface area increase from 96‐well plates (see complete description of the method in Supporting Information). Transgene expression was assayed by luciferase assay, normalized by total protein measured by BCA assay, in units of relative light units per milligram of total protein.

## 3 Results

**Figure 1:**
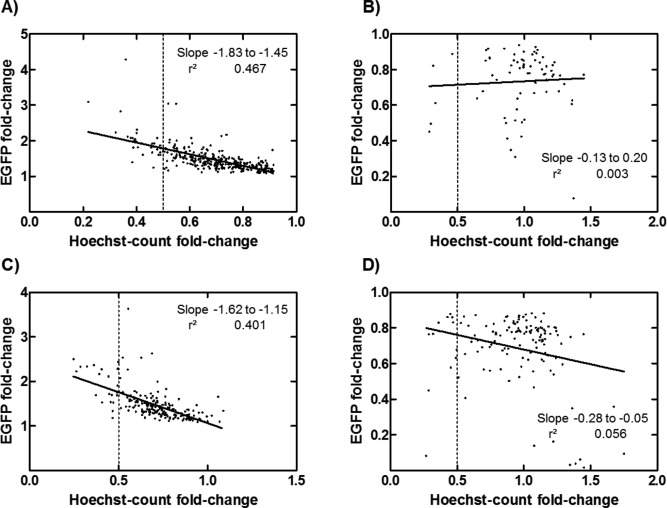
EGFP transfection fold‐changes versus Hoechst‐count fold‐changes are shown for (A) 369 hits with fold‐increases in transfection at 5 µM, (B) 72 hits with fold‐decreases in transfection at 5 µM, (C) 201 hits with fold‐increases in transfection at 50µM, and (D) 132 hits with fold‐decreases in transfection at 50 µM. To examine effect of transfection priming on cell proliferation, linear regressions were performed, demonstrating negative correlation for transfection fold‐increases (AC) and no correlation for transfection fold‐decreases (BD). Regression slopes are displayed as the 95% confidence interval, along with ***R*** ^2^ for goodness of fit. The dashed lines indicate the Hoechst‐count fold‐change threshold of greater than 0.5 used in determining the largest transfection fold‐increases and fold‐decreases of hits in the screen (Tables 1 and 2).

HEK293T cells were primed with 725 compounds from the NCC, transfected with a plasmid encoding for an EGFP reporter protein using 25 kDa branched PEI, and assayed by fluorescence microscope imaging and plate reader measurement of EGFP, Hoechst 33342, and resazurin, to screen for compounds that enhance or decrease transfection, without significant toxicity (see Supporting Information Tables S1–S4 for the EGFP and Hoechst fluorescence intensity and count data of the compounds listed in Tables 1 and 2). With an α‐value for significance of 0.05 for average fold‐changes of duplicates and two toxicity filters, the screen returned 441 priming compound hits at 5 µM with EGFP fold‐change ranging from 0.077 to 4.29, and Hoechst‐count fold‐changes ranging from 0.22 to 1.45 (Figures 1A and 1B). There were 333 hits at 50 µM, with EGFP fold‐change ranging from 0 to 3.63, and Hoechst‐count fold‐changes ranging from 0.25 to 1.75 (Figures 1C and 1D).

At 5 µM, there were 369 hits with transfection fold‐increases (1.45 ± 0.34 fold‐change, mean ± standard deviation) and 72 hits with transfection fold‐decreases (0.73 ± 0.17 fold‐change) (Figures 1A and 1B). At 50 µM, there were 201 hits with transfection fold‐increases (1.47 ± 0.35 fold‐change) and 132 hits with transfection fold‐decreases (0.69 ± 0.20 fold‐change) (Figures 1C and 1D). To identify the most effective priming compounds from the hundreds of hits, the 10 compounds that resulted in the largest EGFP fold‐change, in fold‐increase or fold‐decrease in transfection, with Hoechst‐count fold‐change no less than 0.5, were identified at 5 µM (Table 1) and 50 µM (Table 2).

Linear regressions of EGFP fold‐change versus Hoechst‐count fold‐change were performed to determine correlations between transfection priming and cell proliferation. There were negative correlations between EGFP fold‐increases versus Hoechst‐count fold‐change at both 5 and 50 µM, with *R* ^2^ = 0.467 and 0.401, respectively (Figures 1A and 1C). There were no correlations of EGFP fold‐decreases versus Hoechst‐count fold‐change at either 5 or 50 µM, *R* ^2^ = 0.003 and 0.056, respectively (Figures 1B and 1D).

**Figure 2:**
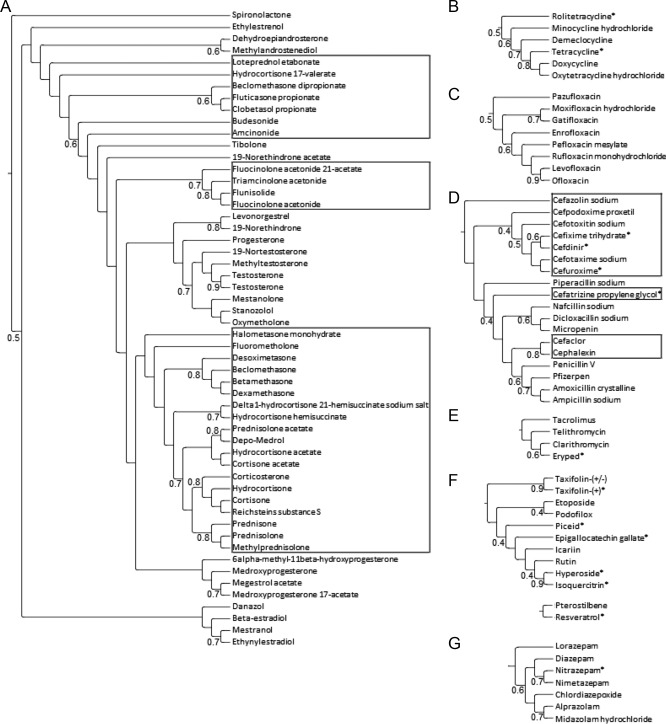
Dendrograms illustrate hierarchical clustering of hormones (A), tetracyclines (B), fluoroquinolones (C), β‐lactams (D), macrolides (E), flavonoids and stilbenoids (F), and benzodiazepine GABAA receptor modulators (G), from the NCC, annotated with Tanimoto coefficients to indicate degree of similarity between compounds in the clusters. Outlined in (A) are glucocorticoids, outlined in (D) are cephalosporins. * indicates compounds which appear in Table 1 or 2.

To further analyze the transfection priming hits, drugs within the NCC were clustered into groups of similar compounds by binning clustering of PubChem fingerprints at Tanimoto clustering coefficient thresholds ranging from 0.4 to 0.9. Cluster number increases as clustering coefficient for similarity is increased, from 300 clusters at clustering coefficient 0.4, to 619 clusters at clustering coefficient 0.9. Based on the identification of compounds that had the most increased or decreased transfection fold‐changes (Tables 1 and 2) clusters of interest were identified (Figure 2), including antibiotics (tetracyclines, β‐lactams, and macrolides), flavonoids, stilbenoids, and GABA receptor modulators. For these clusters of interest, average transfection fold‐changes and average Hoechst‐count fold‐changes were determined (Table 3).

### 3.1 Antibiotics

Six antibiotics showed among the largest fold‐decreases in transfection at 50 µM in the screen, including four cephalosporin β‐lactams and two tetracyclines (Table 2), with transfection fold‐decreases ranging from 0.017 to 0.139, and associated cell‐count and Hoechst‐intensity fold‐changes ranging from 1.08 to 1.75‐fold increase and 0.78 to 0.88‐fold decrease, respectively. One antibiotic, Eryped, was one of the ten compounds that showed the highest fold‐increase in transfection at 50 µM (Table 2), with fold‐changes of 1.99, 0.72, 1.26 and 1.26, in transfection, cell‐count, Hoechst‐intensity, and resazurin‐intensity, respectively.

From the clustering perspective, a majority of hits in the screened β‐lactam, tetracycline, and macrolide clusters were identified as hits that showed a significant fold‐decrease in transfection at 50 µM (Table 3). No antibiotic clusters exhibited a majority of hits decreasing transfection at the 5 µM concentration. A majority of the screened fluoroquinolones and macrolides were identified as hits that produced significant fold‐increase in transfection at 50 µM (Table 3), and a majority of the screened fluoroquinolones, macrolides, and tetracyclines demonstrated significant fold‐increase in transfection at 5 µM (Table 3).

### 3.2 Flavonoids and stilbenoids

Four of the ten compounds that showed the largest fold‐decrease in transfection at 50 µM in the screen are flavonoids (Table 2), with transfection fold‐decreases ranging from 0 to 0.36, and associated cell‐count and Hoechst intensity fold‐changes ranging from 0.8 to 1.68‐fold and 0.86 to 0.87‐fold, respectively. One of these flavonoids, EGCG, also showed 0.08, 1.37, and 0.88 fold‐changes in transfection, cell‐count, and Hoechst‐intensity, respectively, at 5 µM (Table 1). At 5 µM, nobiletin was one of the ten compounds that showed the highest fold‐increase in transfection, with fold‐changes of 2.08, 0.61, 1.23, and 1.21 in transfection, cell‐count, Hoechst‐intensity, and resazurin‐intensity, respectively (Table 1).

Stilbenoids demonstrated among the highest fold‐increases in transfection observed in the screen. Piceid was one of the ten compounds that showed the highest fold‐increase in transfection at 50 µM (Table 2), with fold‐changes of 2.63, 0.69, and 1.17, in transfection, cell‐count, and Hoechst‐intensity, respectively, while resveratrol was one of the ten compounds that showed the highest fold‐increases in transfection at 5 µM (Table 1), with fold‐changes of 3.04 and 0.55, in transfection and cell‐count, respectively.

### 3.3 GABAA receptor modulators

GABAA receptor modulators demonstrated among the highest fold‐increases to transfection in the screen at both 5 and 50 µM. No GABAA receptor modulators were identified as hits for fold‐decreases in transfection. Stiripentol was one of the ten compounds that showed the highest fold‐increase in transfection at 50 µM (Table 2), with fold‐changes of 1.77, 0.72, and 1.23, in transfection, cell‐count, and Hoechst‐intensity, respectively. Zolpidem tartrate and nitrazepam were two of the ten compounds that showed the highest fold‐increase in transfection at 5 µM (Table 1), with fold‐changes of 3.05 and 2.03, and 0.52 and 0.53, in transfection and cell‐count, respectively. From the clustering perspective, a majority of the compounds in the GABAA modulator cluster were hits exhibiting fold‐increases in transfection at 5 µM (Table 3).

### 3.4 Hormones

The hormone cluster had the most member compounds of all clusters in the NCC collection, with 54 members, 30 of which are glucocorticoids. From the clustering perspective, the majority of glucocorticoids and hormones demonstrated fold‐increases in transfection at 5 µM (Table 3). However, hormones were not among the compounds with largest fold‐increases or fold‐decreases in transfection in the screen (Tables 1 and 2).

## 4 Discussion

Nonviral gene delivery methods are not currently competitive with viral transduction in clinical trials.^10^ Pharmaceutical priming has been shown to improve transfection efficiency,^11^, ^12^, ^13^, ^14^, ^15^, ^16^, ^17^, ^18^, ^19^ although in the case of glucocorticoids for example, their priming mechanisms are not well understood. Transfection priming effects of certain compounds are also likely to be cell type and carrier specific, so there is a need to search for more classes of priming compounds to generate a library of transfection priming candidates and better understand tunable priming mechanisms. Preliminary research into transfection priming should seek compounds that have a low development cost and are known to be biocompatible and bioavailable. The particular collection of pharmacological compounds screened was the NCC from the NIH Small Molecule Repository,^20^ which is provided to researchers to repurpose clinically approved pharmaceutical compounds. NCC compounds have known drug‐like properties, with well‐studied bioactivity, making the NCC an ideal starting point for a high‐throughput screen to discover priming compounds for nonviral gene delivery.

### 4.1 Overall screen results

Of the 725 NCC compounds screened for their effect on transfection, hundreds had significant effects on transfection at 5 or 50 µM doses, of which the most effective priming compounds were identified and examined for clustered effects of several drug classes. The highest concentration tested in this screen was 50 μM, which was based on a previous high throughput screen that similarly screened, in vitro, for novel chemoprotective compounds at 50 μM.^24^ Additional concentrations at orders of magnitude lower concentration (5 and 0.5 μM) were considered in order to test for compounds which require more dilute concentrations to affect transfection fold‐changes with minimal cytotoxicity. In preliminary screens carried out at 0.5, 5, and 50 μM in our lab (data not shown), the majority of potential hits were identified at 5 and 50 μM, therefore, these two concentrations were chosen for the complete NCC screen.

It must be emphasized that within this screen, the priming compounds and PEI/DNA complexes were in fact not added at the same time, with the priming compounds delivered to cells 1 hr prior to delivery of the PEI/DNA complexes. This timing was selected based on previous studies,^15^, ^16^, ^17^, ^18^, ^19^ which demonstrated that priming compound effects do not require codelivery with the transfection, and in fact were often most potent when applied in a short window of time, including the 1‐hr time point, prior to transfection. The screen was conducted in duplicate (*n* = 2) with normalization of transfection measures to minimize artifacts.

Preliminary verification experiments were performed for three of the priming compounds identified: resveratrol, corticosterone, and epigallocatechin gallate. Resveratrol and EGCG were chosen to verify antioxidants, a drug class that demonstrated among the largest transfection fold‐changes observed in the screen; furthermore, these two drugs demonstrated opposite effects on transfection, given resveratrol was shown to increase transfection in the screen (3‐fold at 5 μM), while EGCG knocked down transfection (13‐fold at 5 μM) in the screen. Corticosterone was chosen as one of the glucocorticoid hormones in the NCC, a large class of priming compounds that exhibited modest transfection fold‐increases (corticosterone: 1.5‐fold at 5 μM) in the screen. In the verification experiments, these compounds were tested in triplicate at 5 μM, with resveratrol exhibiting approximately 5‐fold increase in transfection relative to control, corticosterone exhibiting approximately 3.5‐fold increase, and EGCG exhibiting 38‐fold decrease; these further experiments agree with the screen results (see Supporting Information Figures S1 and S2).

There was a negative correlation in screen hits between transfection fold‐increases and cell‐count fold‐change, which may be related to interference of priming compounds with normal cell processes, or increased toxicity of EGFP as it is overproduced due to priming. Drug clustering results indicate that certain compounds may be modulating the cell response to PEI transfection; those clusters include antibiotics, antioxidants, GABAA modulators, and glucocorticoids, as discussed next.

### 4.2 Antibiotics

From the results of the screen, antibiotic priming compounds were shown to affect transfection, with many showing potent inhibition (Table 2). Many protocols recommend transfection in medium without antibiotics, as they are thought to decrease transfection or cell viability.^25^, ^26^ However, among the antibiotics in the NCC, antibiotic effects on transfection were observed to be dose and antibiotic‐class dependent. At 50 µM, the majority of cephalosporin‐ and tetracycline‐class antibiotics screened resulted in large fold‐decreases in transfection (Table 3), while the fluoroquinolone‐class did not show transfection fold‐decreases at 50 µM. At 5 µM, the majority of antibiotics in the fluoroquinolone‐, macrolide‐, and tetracycline‐class clusters resulted in transfection fold‐increases; the majority of β‐lactam/cephalosporin cluster antibiotics did not prime transfection fold‐increases at 5 µM.

The mechanisms that cause the transfection priming effects of antibiotics observed in the screen may be related to the primary off‐target of antibiotics in mammalian host cells, which is mitochondria.^27^ Modulation of mitochondrial dysfunction and oxidative stress response could explain the effects antibiotics have on transfection, particularly when utilizing transfection reagents that are known to affect mitochondria, such as PEI. PEI has been shown to cause mitochondrial dysfunction through disruption of the mitochondrial membrane that can result in oxidative stress and apoptosis.^28^ If the identified antibiotic classes are inducing their priming effects through interaction with mitochondria, it would be evidence that PEI‐induced mitochondrial dysfunction and associated autophagy can be modulated to either increase or decrease transfection by design of the priming compound.

### 4.3 Flavonoid and stilbenoid compounds

The priming effects observed in antioxidants tested from the NCC in this screen provide further evidence that modulation of mitochondrial dysfunction is a potent priming mechanism of PEI transfection. The flavonoid nobileton showed 2‐fold increase in transfection at 5 µM, among the largest in the screen. Conversely, several flavonoids were among compounds with largest fold‐decrease in transfection at 50 µM; EGCG exhibited the most potent fold‐decrease in transfection of the screen at both 5 and 50 µM, with fold‐decreases of 0.08 and 0, and cell‐count fold‐changes of 1.37 and 0.8, respectively (Tables 1 and 2). The stilbenoids resveratrol and piceid, at 5 and 50 µM, respectively, exhibited nearly 3‐fold increases in transfection, which were among the largest seen in the screen.

Like antibiotics, antioxidant transfection priming effects observed in the screen can potentially be explained by interactions with mitochondria, however, in a manner that generally protects or improves their function.^29^ Resveratrol is a natural phenol, specifically a stilbenoid, with antioxidant properties, that can be found in grape skins.^29^ Piceid is a another plant‐derived stilbenoid, specifically a glucoside modification of resveratrol. Resveratrol has been shown to rescue mitochondrial dysfunction and modulate autophagy,^30^ which could be beneficial by mitigating the cytotoxic effects of PEI transfection,^28^ from PEI's toxic interactions with mitochondria, to oxidative stress induced by transgene expression.^16^, ^17^, ^18^, ^19^, ^31^ If antioxidants are modulating transfection through mitochondria and oxidative stress response,^32^ the observed antioxidant priming effects that potently increase or decrease transfection provide additional evidence that the cellular stress response to transfection is critical to overall transfection outcome,^16^, ^17^, ^18^ a response that is tunable by priming.

### 4.4 GABAA receptor modulators

Like antibiotics and antioxidants, GABAA modulators were also identified by the screen as compound clusters in the NCC that resulted in large transfection fold‐changes, with the majority of GABAA modulators (seven of nine compounds) increasing transfection at the 5 µM concentration. Zolpidem, nitrazepam, and stiripentol were identified to cause among the highest transfection fold‐increases in in the screen: 3, 2, and 1.7‐fold, respectively (Tables 1 and 2).

GABAA receptors are neurotransmitter receptors that are not expressed in most cell types, so the mechanism behind priming effects seen with the GABAA modulator cluster is not immediately apparent. Benzodiazepines, stiripentol, and zolpidem are positive allosteric modulators of the GABAA receptor found in neurons, however, they are thought to act on different subunits.^33^, ^34^ The GABAA receptor, or some of the subunits, may be expressed in HEK293T cells, a plausible possibility due to the proposed adrenal cortex origin of the HEK293T cell line,^35^ which may explain the observed transfection priming effects of GABAA modulators in the screen, although the potential mechanism of interaction between GABAA receptor modulation and transfection is not clear.

Another possibility that may explain the transfection priming effects of GABAA modulators is interaction with the translocator protein, also known as the peripheral benzodiazepine receptor, which is found in mitochondria membranes, and is thought to be involved in steroidogenesis and redox regulation of mitophagy.^36^, ^37^ This interaction would be consistent with the concept of modulating mitochondrial dysfunction and cellular stress response to transfection as the mechanism by which priming is occurring through antibiotics and antioxidants (see above).

### 4.5 Glucocorticoids and other hormones

Finally, the effects of glucocorticoids and other hormones in the NCC were examined, as glucocorticoids have previously shown enhancement of lipoplex‐mediated transfection in hMSCs by over 10‐fold.^15^ The hormone cluster was the largest cluster of drugs in the NCC, and the majority of these hormones were glucocorticoids (Figure 2). In this screen, the majority of hormones, and glucocorticoids specifically, increased transfection at the 5 µM concentration, with average ∼1.4‐fold increases in transfection. This transfection fold‐change is much less than the 10‐fold increases seen previously in hMSCs^15^ indicating priming effects for at least certain compounds are dependent on cell type and/or transfection reagent.

There are several plausible explanations for the transfection priming fold‐increases observed in glucocorticoids and other hormones in this screen. Glucocorticoids are hormones that are involved in the body's stress response, activating the glucocorticoid receptor, which translocates to the nucleus and acts as a transcription factor.^15^ Upregulation of certain genes by activated hormone receptors in response to priming by hormones may play a role in the observed transfection fold‐changes in the screen, as it has previously been shown in gene expression profiling studies that pharmacological priming of genomic targets can affect transfection.^16^, ^17^, ^18^, ^19^ Activated glucocorticoid receptors may also play a role in priming transfection by assisting internalization, nuclear transport, and nuclear import of transfection complexes. Finally, glucocorticoid priming effects in PEI transfection could be occurring through interaction with mitochondria and autophagy,^38^, ^39^ which would be further evidence that aligns with the hypothesis that modulation of PEI transfection toxicity and cellular stress response are the priming mechanisms for antibiotics, antioxidants, and GABAA clusters.

### 4.6 Mitochondrial dysfunction, oxidative stress, and autophagy in PEI transfection

PEI is a cationic polymer carrier for nonviral gene delivery that serves as a standard to which newly developed carriers are often compared, as it reliably overcomes the primary barriers to transfection of cultured mammalian cells: internalization, endosomal escape, and nuclear transport,^8^ although not with the efficiencies found in viral vectors.^9^ Paradoxically, PEI designs that are more effectively able to overcome barriers to transfection also tend to be more toxic to the transfected cell. For instance, high molecular weight (MW) and branched PEI complexes have been demonstrated to transfect at higher efficiencies compared to low MW and linear PEI complexes,^40^, ^41^ but also exhibit higher toxicity. Attempts to resolve this dilemma include bioresponsive linkages of low MW PEI into higher MW PEI‐based polymers that will degrade in the cell into less toxic lower MW constituents.^40^

PEI has been shown to be cytotoxic through its accumulation in and interaction with mitochondrial membranes.^28^ Damage to mitochondria has been implicated in the intracellular inflammatory and immune response^42^, ^43^; PEI has been shown to be an effective adjuvant for stimulating an immune response.^44^ Typical cellular responses to mitochondrial damage are autophagy/mitophagy, apoptosis, and necrosis. Apoptosis and autophagy are mutually inhibitory,^45^ with apoptosis leading to cell death, while autophagy possibly serves a protective role in PEI cytotoxicity.^46^

Altogether, literature review of grouped screen hit compounds suggests that many priming effects may be due to modulation of the cellular oxidative stress response to PEI transfection, in particular mitochondrial dysfunction. It is plausible that certain antioxidants like resveratrol^29^, ^30^ rescue the cell from PEI‐induced mitochondrial dysfunction through modulation of autophagy, improving transfection, while certain antibiotics that reduce transfection, such as cephalosporins,^47^ may be inhibiting transfection by increasing mitochondrial dysfunction and oxidative stress.

Gene expression profiling has shown that there is differential expression in the cellular oxidative stress response associated with successfully versus unsuccessfully PEI‐transfected cells, through ATF3, IREB2, and other gene pathways.^16^, ^17^, ^18^, ^19^ The high‐throughput screen data in this current study support this finding, and suggest that modulating the cellular stress response to PEI transfection by priming with clinically approved compounds prior to PEI transfection can significantly affect overall transfection efficiency and transgene expression in vitro.

## 5 Conclusions

With a high‐throughput, drug‐repurposing, and bio/cheminformatics clustering approach, this screen of the NCC identified many individual compounds that produce dramatic fold‐changes in HEK293T transfection by PEI, and several classes of clinical drugs for which the majority of compounds demonstrated priming effects. These hit compounds and drug clusters should be verified and studied for investigation of the priming mechanisms in detail that will improve our understanding of the cellular response to transfection and how this response can be pharmacologically modulated to improve transfection. The clustered data suggest that several of these drug classes, such as stilbenoids and flavonoid antioxidants, cephalosporin antibiotics, GABAA receptor modulators, and others, may be priming transfection through modulation of mitochondrial dysfunction, oxidative stress, and cell death processes caused by toxicity of PEI transfection. These compounds and drug classes should be tested on additional cell types with alternative nonviral carriers to generalize the priming effects towards potential application as transfection adjuvants. For future in vivo studies, the 5 and 50 μM concentrations can serve as starting points in the optimization of the delivery method and dose and release characteristics of each priming compound to maximize transfection priming effects. Additionally, timing of the addition of the priming compound relative to the DNA should be studied more extensively as a tunable priming parameter. Resveratrol and zolpidem had the highest fold‐change increases observed in the screen, at 3‐fold; the potency of their priming effect indicates their mechanisms may be critical to understanding how nonviral gene delivery can be improved by priming.

## Supporting information

Supporting Information

Supporting Information
