# Supplementary material for: High‐throughput screening of clinically approved drugs that prime polyethylenimine transfection reveals modulation of mitochondria dysfunction response improves gene transfer efficiencies
Source: Bioeng Transl Med. 2016 Jul 21;1(2):123–35. doi: 10.1002/btm2.10017 (PMC5127179; doi:10.1002/btm2.10017)
Supplement: Supplementary file 1 — Supporting Information [file BTM2-1-123-s001.docx]

Preliminary verification experiment: HEK293T cells were detached from culture flasks by EDTA, counted by hemocytometer, and seeded at 25,000 cells/cm^2^ (20,000 cells per well in 200 μL) into twelve wells of a 48 well plate for triplicate testing of three compounds (resveratrol, epigallocatechin gallate, and corticosterone) at 5 μM against DMSO vehicle control. Seeded plate was cultured at 37 °C, 5% CO_2,_ and approximately 17 hours after seeding, 100uL of each of the three compounds were delivered into three wells of the plate, at 5 μM final well volume concentration. Three of the wells received equivalent DMSO % instead of tested priming compound as vehicle controls. The primed plate was then incubated for one hour at 37 °C, 5% CO_2_ before transfection. For transfection PEI was diluted in 1X TBS and added to pEGFPLuc diluted in 1X TBS. Complexes were formed at an optimized (data not shown) N:P of 15 to deliver 0.425 μg of pEGFPLuc in 52.5 μL of TBS to each well one hour after priming, followed by incubation at 37 °C, 5% CO_2_ for 48 hours. After the 48 hour incubation, each well was aspirated of media, rinsed with PBS, then lysed with 200 μL of 1X Reporter Lysis Buffer (Thermo Fisher Scientific), incubated at room temperature for 5 minutes then frozen at -80 °C. Transfection levels were quantified by measuring the luciferase activity using the Luciferase Assay System (Promega, Madison WI) and a luminometer (Turner Designs, Sunnyvale, CA). Luciferase activity (measured as relative light units, or RLUs) was normalized to the total protein amount determined with a Pierce BCA protein assay (Pierce, Rockford, IL).
