# Supplementary material for: High‐throughput screening of clinically approved drugs that prime polyethylenimine transfection reveals modulation of mitochondria dysfunction response improves gene transfer efficiencies
Source: Bioeng Transl Med. 2016 Jul 21;1(2):123–35. doi: 10.1002/btm2.10017 (PMC5127179; doi:10.1002/btm2.10017)
Supplement: Supplementary file 2 — Supporting Information [file BTM2-1-123-s002.docx]

Figure S1. Results of the experiment to verify in triplicate (n=3) the transfection priming effects observed in the screen for corticosterone (1.5 fold-increase), resveratrol (3 fold-increase), and epigallocatechin gallate (13 fold-decrease), at the 5 μM concentration. The results of this experiment agree with those screen results, exhibiting 5-fold and 3-fold increases in transgene expression for resveratrol and corticosterone, respectively, and 38-fold decrease for epigallocatechin gallate. Transgene expression was assayed by luciferase assay, normalized by total protein measured by BCA assay, in units of relative light units (RLU) per milligram of total protein. Note: These experiments were carried out in 48 wells plates instead of 96 well plates of the screen, with number of seeded cells and volumes of reagents added scaled by the surface area increase (see description of method in supplemental materials).


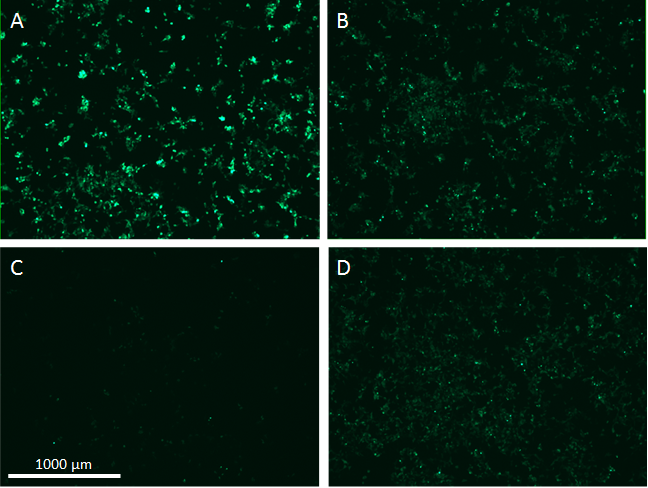


Figure S2. Representative microscope images of EGFP fluorescence images in wells from the transfection priming compound verification experiments summarized in Figure S1, A) resveratrol B) corticosterone C) epigallocatechin gallate D) transfected vehicle control.

| Table S1. EGFP intensity (plate reader relative fluorescence units (RFU) and fluorescence image mean 8-bit grey value), EGFP count, and Hoechst count data for priming compounds identified in screen of NCC (largest fold-increases at 5μm) | | | | | | | | |
| --- | --- | --- | --- | --- | --- | --- | --- | --- |
| Largest fold-increase hits at 5μM (See Table 1) | Plate Reader | | Fluorescence Image Processing | | | | | |
|  | EGFP intensity (RFU) | | EGFP intensity (0-255) | | EGFP Count | | Hoechst Count | |
|  | Replicate 1 | Replicate 2 | Replicate 1 | Replicate 2 | Replicate 1 | Replicate 2 | Replicate 1 | Replicate 2 |
| Zolpidem tartrate | 14370 | 13975 | 14.664 | 16.601 | 3040 | 3372 | 10902 | 6692 |
| Resveratrol | 18203 | 5581 | 14.041 | 29.281 | 4661 | 1850 | 4926 | 12674 |
| Tropisetron hydrochloride | 7937 | 8690 | 8.722 | 9.397 | 3896 | 3037 | 26010 | 26194 |
| Tranilast | 21014 | 15779 | 17.289 | 24.81 | 4585 | 4304 | 14161 | 12047 |
| Lansoprazole | 16113 | 11548 | 14.48 | 15.046 | 2608 | 3939 | 15992 | 8450 |
| Nobiletin | 12454 | 10648 | 13.933 | 12.076 | 2405 | 4315 | 12280 | 8228 |
| Nitrazepam | 6848 | 9235 | 9.285 | 14.009 | 3746 | 1836 | 5938 | 11370 |
| Enalaprilat | 10717 | 9370 | 12.24 | 10.886 | 2740 | 4110 | 11378 | 7285 |
| Droperidol | 10696 | 7558 | 11.854 | 10.562 | 2204 | 3855 | 11878 | 5939 |
| Mestanolone | 10264 | 11598 | 11.463 | 14.457 | 3605 | 3805 | 10430 | 9990 |

| Table S2. EGFP intensity (plate reader relative fluorescence units (RFU) and fluorescence image mean 8-bit grey value), EGFP count, and Hoechst count data for priming compounds identified in screen of NCC (largest fold-decreases at 5μm) | | | | | | | | |
| --- | --- | --- | --- | --- | --- | --- | --- | --- |
| Largest fold-decrease hits at 5μM (See Table 1) | Plate Reader | | Fluorescence Image Processing | | | | | |
|  | EGFP intensity (RFU) | | EGFP intensity (0-255) | | EGFP Count | | Hoechst Count | |
|  | Replicate 1 | Replicate 2 | Replicate 1 | Replicate 2 | Replicate 1 | Replicate 2 | Replicate 1 | Replicate 2 |
| Epigallocatechin gallate | 622 | 2982 | 7.206 | 6.27 | 183 | 875 | 19049 | 22696 |
| Ampiroxicam | 2927 | 3346 | 6.249 | 6.279 | 516 | 291 | 24339 | 25752 |
| Nimodipine | 2858 | 3525 | 6.21 | 6.508 | 735 | 226 | 23040 | 25290 |
| (-)-Cotinine | 2746 | 3845 | 6.105 | 6.665 | 1059 | 135 | 23407 | 23472 |
| Ramipril | 3297 | 3325 | 6.299 | 6.507 | 622 | 526 | 26704 | 28921 |
| Desloratadine | 3119 | 3870 | 6.317 | 6.681 | 940 | 350 | 22101 | 24335 |
| Crotamiton | 2985 | 4586 | 6.178 | 7.368 | 1656 | 128 | 24468 | 28400 |
| Guanidine | 3356 | 3370 | 6.293 | 6.305 | 653 | 582 | 23411 | 26784 |
| Letrozole | 2989 | 4363 | 6.304 | 7.103 | 1403 | 281 | 25879 | 27969 |
| Fluphenazine hydrochloride | 3254 | 4079 | 6.213 | 6.778 | 1007 | 341 | 23056 | 22741 |

| Table S3. EGFP intensity (plate reader relative fluorescence units (RFU) and fluorescence image mean 8-bit grey value), EGFP count, and Hoechst count data for priming compounds identified in screen of NCC (largest fold-increases at 50μm) | | | | | | | | |
| --- | --- | --- | --- | --- | --- | --- | --- | --- |
| Largest fold-increase hits at 50μM (See Table 2) | Plate Reader | | Fluorescence Image Processing | | | | | |
|  | EGFP intensity (RFU) | | EGFP intensity (0-255) | | EGFP Count | | Hoechst Count | |
|  | Replicate 1 | Replicate 2 | Replicate 1 | Replicate 2 | Replicate 1 | Replicate 2 | Replicate 1 | Replicate 2 |
| Tranilast | 23120 | 21250 | 19.829 | 21.273 | 2449 | 3393 | 10300 | 6703 |
| Piceid | 18285 | 20123 | 15.845 | 19.893 | 2204 | 3300 | 15900 | 6301 |
| 5-Fluorocytosine | 22353 | 19025 | 12.662 | 11.529 | 1529 | 1941 | 5748 | 4944 |
| Cinanserin | 3675 | 9635 | 6.878 | 19.804 | 3974 | 406 | 3876 | 12001 |
| Zardaverine | 19725 | 20075 | 13.315 | 14.615 | 2060 | 1922 | 4642 | 6637 |
| Nateglinide | 11802 | 10027 | 12.309 | 12.343 | 1855 | 2532 | 9669 | 6013 |
| Eryped | 14906 | 15360 | 12.837 | 14.81 | 4760 | 5027 | 21440 | 15143 |
| Mestinon | 13272 | 15942 | 11.662 | 13.813 | 4342 | 4921 | 24149 | 15157 |
| Acyclovir | 13614 | 13067 | 13.74 | 11.815 | 3309 | 2848 | 8864 | 10196 |
| Stiripentol | 5060 | 16331 | 20.75 | 15.884 | 2158 | 3620 | 9223 | 7332 |

| Table S4. EGFP intensity (plate reader relative fluorescence units (RFU) and fluorescence image mean 8-bit grey value), EGFP count, and Hoechst count data for priming compounds identified in screen of NCC (largest fold-decreases at 5μm) | | | | | | | | |
| --- | --- | --- | --- | --- | --- | --- | --- | --- |
| Largest fold-decrease hits at 50μM (See Table 2) | Plate Reader | | Fluorescence Image Processing | | | | | |
|  | EGFP intensity (RFU) | | EGFP intensity (0-255) | | EGFP Count | | Hoechst Count | |
|  | Replicate 1 | Replicate 2 | Replicate 1 | Replicate 2 | Replicate 1 | Replicate 2 | Replicate 1 | Replicate 2 |
| Epigallocatechin gallate | 303 | 2286 | 5.984 | 6.049 | 2 | 2 | 21304 | 9259 |
| Cefixime trihydrate | 2929 | 2053 | 5.982 | 6.235 | 86 | 58 | 15048 | 14636 |
| Cefdinir | 3180 | 3610 | 5.984 | 6.425 | 199 | 54 | 13242 | 14277 |
| Cefuroxime | 3036 | 2665 | 6.094 | 6.167 | 271 | 197 | 17142 | 22996 |
| Rolitetracycline | 3929 | 2323 | 6.424 | 6.59 | 277 | 257 | 15541 | 13560 |
| Cefatrizine propylene glycol | 2746 | 3889 | 6.33 | 6.661 | 594 | 414 | 16191 | 16249 |
| Tetracycline | 3731 | 2889 | 6.79 | 6.476 | 572 | 872 | 17861 | 22680 |
| Taxifolin-(+) | 5795 | 3854 | 7.295 | 6.958 | 439 | 898 | 12469 | 12494 |
| (+/-)-Epinephrine hydrochloride | 12441 | 3487 | 11.807 | 6.554 | 539 | 3546 | 18073 | 21152 |
| Hyperoside | 7955 | 7007 | 11.671 | 7.797 | 1637 | 3898 | 21156 | 17253 |
